# INTEREST GROUP SESSION—CANCER AND AGING: CURRENT CHALLENGES IN CANCER SCREENING AMONG OLDER MINORITY POPULATIONS

**DOI:** 10.1093/geroni/igz038.678

**Published:** 2019-11-08

**Authors:** Chien-Ching Li, Darren Liu

**Affiliations:** 1 Rush University, Chicago, Illinois, United States; 2 Des Moines University, Des Moines, Iowa, United States

## Abstract

Cancer is an important public issue around the world. Among types of cancer, lung and colorectal cancer are the most common in men while breast and cervical cancer are the most common in women. Detection of early stage cancer via screening can significantly reduce the mortality and prolong life. Although cancer prevention and control has been served as the national priority, individual’s utilization of cancer screening services is low due to limited knowledge of cancer screening and ineffective patient-provider commutation, especially in minority populations. In this symposium, we will examine three scenarios that highlight the challenges of cancer screenings in minority populations. First, we will share the results from a mixed method study that investigate the knowledge and attitudes towards Low Dose Computed Tomography lung cancer (LDCT) screening and assess the smoking cessation needs for African Americans who receive LDCT screening in an effort to reduce the health burden of lung cancer. The next study will discuss how the characteristics of older Chinese adults from the United States and Taiwan are associated cancer screening communication with physicians (i.e., whether doctor recommended screenings and whether communicated screenings with doctor). Lastly, we will share the results from a cross-sectional study that analyzed 10 years data of National Health Interview Survey to examine the difference in LDCT screening eligibility among Asian American (i.e., Chinese, Filipino, and other Asian) smokers. The discussant will summarize with an overview of the topic, and comment on the disparities of cancer screening in older minority populations.

